# Consenting of the vulnerable: the informed consent procedure in advanced cancer patients in Mexico

**DOI:** 10.1186/1472-6939-7-13

**Published:** 2006-12-13

**Authors:** Emma L Verástegui

**Affiliations:** 1 Ethics Committee, Instituto Nacional de Cancerologia, Avenida San Fernando 22, Mexico City, Mexico; 2 Comision Nacional de Bioetica, Torre Zafiro 1, Periferico Sur 4118, piso 1, México City, Mexico

## Abstract

**Background:**

A topic of great concern in bioethics is the medical research conducted in poor countries sponsored by wealthy nations. Western drug companies increasingly view Latin America as a proper place for clinical research trials. The region combines a large population, modern medical facilities, and low per capita incomes. Participants from developing countries may have little or non alternative means of treatment other than that offered through clinical trials. Therefore, the provision of a valid informed consent is important.

**Methods:**

To gain insight about some aspects of the informed consent procedure in a major cancer centre in Mexico, we conducted a three-step evaluation process: 1) a ten point multiple choice survey questionnaires, was used to explore some aspects of the patients' experiences during the informed consent process, 2) researchers' knowledge about specific aspects of the informed consent was evaluated in this study using survey questionnaires; and 3) the comprehensibility, readability and number of pages of the consent forms were analysed. The socioeconomic and educational level of the patients, were also considered. Results were reported using a numerical scale.

**Results:**

Thirty five patients, 20 doctors, and 10 individuals working at the hospital agreed to participate in the study. Eighty three percent of the patients in the study were classified as living in poverty; education level was poor or non existent, and 31% of the patients were illiterate. The consent forms were difficult to understand according to 49% of the patients, most doctors agreed that the forms were not comprehensible to the patients. The average length of the IC documents analysed was 14 pages, and the readability average score was equivalent to 8^TH ^Grade.

**Conclusion:**

The results presented in this work describe some relevant characteristics of the population seen at public health care institutions in Mexico. Poverty, limited or no education, and the complexity of the information provided to the patients may question the validity of the informed consent procedure in this group of patients.

## Background

All achievements of modern medicine stem from research. Advances, now taken for granted, were developed through experimentation, which was conducted, for the most part, according to the standards and theories available at the time that it was conducted [1]. Even though guidelines and rules have been implemented, the setting in which research takes place is constantly changing, and sometimes both conducting research and choosing not to conduct it can be morally problematic [2-6].

Currently, a topic of great concern in medical ethics is the biomedical research being sponsored by wealthy nations and conducted in underprivileged countries. Unequal resource distribution between external research sponsors and health care facilities in developing countries may increase the hazard of exploitation [7].

As has been reported for HIV/AIDS clinical trials in Africa, developing countries may have different motivations to take part in a clinical trial as prospective participants may have little or no alternative means of receiving health care for their conditions, other than that offered through clinical trials; therefore, enforcement of ethical research procedures represents a major goal for clinical research [7-13].

The 'consent procedure' is associated with the origins of bioethics and is considered that it is a necessary, but not sufficient, condition for research on a subject to be ethical [14-18].

The rationale of the IC according to some, is based on the legal and ethical right that a patient has to be fully informed before he/she accepts what happens to his/her body; and from the ethical duty that physicians have to involve patients in the decision making process regarding their treatment. However, according to others, the IC is the result of changes in the traditional relations of trust [18].

The validity of the informed consent assumes an independent, competent [19] individual, whom freely gives a, reasoned approval for a given procedure [20-22]. The patients' right to be fully informed is increasingly heralded as the ethical panacea, preventing the potential danger of paternalistic autocratic practices [15].

There are many distinct conceptions of individual autonomy, and their ethical importance varies; ethics committees in the West consider the individual autonomy the main moral principle in the decision-making process of the patients; its influence tends to over-ride the value of other bioethical principles [18-21]. In contrast, ethical systems in non-Western cultures may be less dialectical, less analytical, and more sensitive to family or community consensus than to individual autonomy [22-25].

A central point in either scenario is the need to comprehend the information received before an independent consent is given; consequently, to ensure a sufficient level of understanding of the procedure represents an important challenge during the informed consent procedure.

Experimental cancer treatments require special considerations from different perspectives; many treatments are expensive, and often require to be given for long periods of time. Frequently, during the course of the disease, standard approved treatments are exhausted; therefore, most cancer patients will eventually participate in experimental clinical trials [26,27].

For patients living in most developing countries, the absence of universal health care coverage results in complex difficulties to access expensive medical treatments; in consequence, front line cancer chemotherapy may not be available for an important number of patients, except if enrolled in a clinical trial [12,28,29].

Nowadays, clinical studies for cancer treatment world-wide are mostly funded by pharmaceutical companies [30,31]. At the Instituto Nacional de Cancerología (INCan), industry-funded trials account for more than fifty percent of research activities, hence, the population attending this institution benefits from treatments otherwise not available to them [32]. These patients' economic, social, and educational characteristics, as well as their medical condition, dependency situation, and desperation for any treatment that may offer them any hope, are paradigmatic characteristics of a vulnerable population [33].

The value and importance of a valid informed consent (in any research involving human beings, and these patients in particular) is without question. In Mexico, the need of an informed consent for patients involved in clinical research is required by law; in research sponsored by international pharmaceutical companies, a careful monitoring of the informed consent procedure is done by international Clinical Research Organizations (CRO), according to the request of the authorities from the countries of the different sponsors [34,35].

The informed consent forms given to patients enrolled in these research studies are standard forms provided by the sponsor companies. Often, the studies are multicenter trials, or have been previously undertaken in developed countries; therefore, the consent forms are multipage, translated documents, with uniform criteria for all the centers where the trial is being conducted. The IC procedure, through complex documentation, fulfills ethical, regulatory and legal requirements, both for Mexican and International authorities. For the international sponsored clinical research, clinical research organisations (CROs) have an important role making sure that the informed consent procedure is applied according to Good Clinical Research Practices. However, non conclusive information suggest that research participants frequently, may not understand the information presented during the informed consent procedure; therefore, different methodologies to improve understanding during the IC procedure have been tried, however, further research is needed [36-38].

The purpose of this paper is to assess some aspects of the IC procedure from different perspectives in order to detect possible ways to improve the procedure in the near future, including: 1) experiences regarding the IC of a group of patients enrolled in clinical research trials, 2) doctors' perception about the consent procedure, of international sponsored trials and 3) review of several features from randomly selected consent forms.

## Methods

With the purpose of assessing clinical research participants' experiences during the informed consent process in the setting of a cancer centre in Mexico; we conducted a prospective analysis of some aspects of the procedure. The study design included the evaluation of ten different consent forms provided by international sponsors and the use of questionnaires to evaluate the informed consent (IC) procedure for patients enrolled in clinical research trials. The protocol had been approved by the hospital Ethics Committee, requiring only a verbal consent for participation of the different subjects in the study.

### Patients

During a three week period, patients present for routine follow up at the outpatient clinic of the hospital were invited to participate in the survey; after informing the purpose and nature of the study, a verbal consent was obtained. All the participating patients received a short letter, which was read with them (Additional file 1); and a ten question printed survey was applied. After explaining the procedure, free alone time or in the company of their family was provided to answer the questionnaire (Additional file 2).

Survey questionnaires included selected responses to statements (yes or no) and/or multiple options for answers. Participants were able to select more than one statement and to add comments to selected questions.

Socio-economic and educational level of the patients was established. A six point evaluation program (family income, occupation, nutrition, living conditions, place of residence and presence of sick people within the family) used by social workers on all patients admitted to the INCan. The results of the social workers' evaluation, and some medical and demographic characteristics, were obtained from medical records.

Results were expressed numerically, as well as a percentage, along with relevant characteristics presented in the results.

### Doctors

Doctors were invited to participate through personal communication. During the initial approach, the purpose of the study was explained, in addition a description was sent to them by e-mail. In a second personal interview, they were given the option of answering the questionnaire either in a printed or electronic version. Consent for participation was verbal and a three week period was provided before collecting the forms (Additional file 3).

### Informed consent documents

A critical reading of ten different consent forms sent to be approved by the Ethics Committee of the Instituto Nacional de Cancerología was done by a single rater; the documents were read both in English and their translated versions in Spanish. Several points were individually assessed in all the documents: 1) number of pages, 2) fulfilment of the basic elements of informed consent (explanation of the purpose of the research, expected duration, risks, benefits, alternative procedures, confidentiality, compensations, contact person, statement of voluntary participation), 3) original language of the CF (the first letter of the original language of the document was recorded, i.e. 'E" for English). A visual numerical scale evaluation was done by a single rater (there is no inter-rater reliability data) for the assessment of the following aspects: 4) accuracy of the translation, 5) use of medical or scientific terms, 6) use of language not used by non-medical individuals, and 7) medical language that may be difficult to understand by average individuals seen at the institution. Results represent averages of scores (for question 4, 1 was poor and 10 was good and for questions 5, 6, and 7, 1 meant few and 10 many), and 8) readability score was assessed according to sample paragraphs published by Paasche-Orlow and based on the Flesch–Kincaid readability scale, the method developed by Fry [39,40] (Additional file 4 (part A))

The comprehensibility of the consent form was also qualitatively scrutinized with the help of ten individuals: two nurses, two secretaries, three ambulance drivers, two social workers, and one medical resident. Selected consent forms were read by these individuals after the sponsor's name and study drug(s) were blanked out. A standard set of questions were asked after reading the forms; the responses by most of the individuals are described in Additional file 4 (part B).

## Results

### Patients

An invitation letter previously approved by the Ethics Committee was given to fifty patients (Additional file 1), of which only 35 agreed to participate. Most of the patients had been attending the hospital for two to five years. All of the patients had advanced cancer (data not shown) and failed to respond to standard therapy; and were participating in Phase II or III clinical trials involving chemotherapy, radiotherapy, and immunotherapy or targeted therapies.

All the patients were or had been enrolled in trials financed by international pharmaceutical companies. The studies comply with all of the requirements of health and hospital authorities.

### Socioeconomic factors

**Figure 1:**
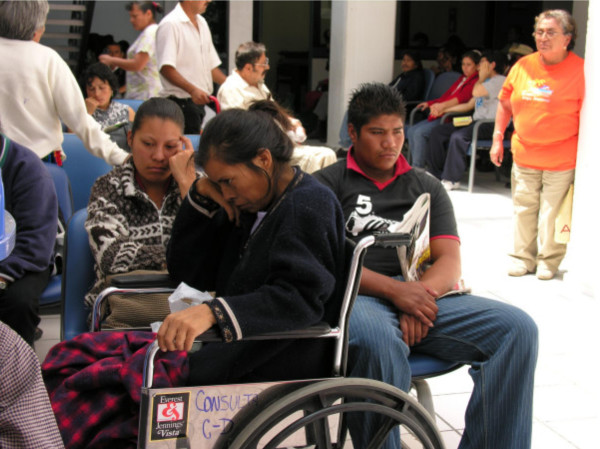
**Patients' population**. Any circumstances in which and individuals are in a position of dependency (research subjects, elderly, indigenous, poor people, dying patients) makes them potentially vulnerable.

A review of participating patients' medical records showed that most of them lived in rural or low income urban areas. Eighty-three percent of the patients were classified as living in poverty, based on a six point socioeconomic evaluation (see Figure 1). The income of most of the patients admitted to the hospital was found to be less than one to two times the minimum wage, $4.00 U.S./day and seven of the enrolled patients were living on less than one dollar per day. Their educational level was also established to be poor, 31% of the patients were illiterate, 29% did not finish grade school, only 20% attend up to grade school, and seven (20%) had additional education (Table 1).

**Table 1:** Characteristics of the Patients in the Study

Characteristic	Number	Average
Number of Patients	35	
Age (yrs)		
Median (range)	59 (27–92)	
Mean	57.9	
Gender		
Female	20	
Male	15	
Schooling		
None	11	31
Grade School Incomplete	10	29
Grade School Complete	7	20
High School Incomplete	4	11
High School Complete	3	9
College/University	0	0
Place of Residence		
Urban	11	31
Rural	24	69
Average Family Income		
Less than 1 minimum wage	12	34
2–5 minimum wage	15	43
More than 5 minimum wages	8	23
Tumor Site**		
Head and Neck	8	23
Prostate	4	11
Malignant Melanoma	6	17
Breast Cancer	8	23
Cervical Carcinoma	9	26

*Minimum wages are slightly different in rural and urban areas; however, it is approximately $4 U.S. Dollars per day. Less than one minimum wage corresponds to extreme poverty, from 2 to 5 minimum wages are classified as poor people. Cervical Cancer patients often live in extreme poverty.

**All patients had advanced cancer.

### Enrolment into the clinical trials and IC procedure

All the patients had been undergoing treatment at the institution for more than two years; most of them had had several doctors in charge of their treatment. At the time of enrolment in the clinical trial, their treating physician had invited them to participate (86%). Their physician explained about the treatment in all the cases and asked them to read and sign the informed consent, always opened to questions. After signing the informed consent, only two patients knew what the document explained about the treatment risks and benefits (6%). In response to the specific question about why they were asked to sign the informed consent, 16 of 35 patients (46%) answered "to receive free treatment", 29% responded that it was to free the hospital and doctors of any responsibility. Interestingly enough, none of the patients thought that the purpose of the consent was to protect their rights as a patient, even though this answer was part of the questionnaire (Table 2).

**Table 2:** Patients' Perceptions about the Informed Consent

Question	Number	%
Who invited you to participate in the clinical trial?		
My treating physician	30	86
A "new" doctor	4	11
A resident	1	3
Were you asked to read and sign an informed consent?		
Yes	35	100
No	0	0
Do you know what a consent form is?		
A document part of the protocol	23	65
A document that I have to sign before I can be admitted to the study	10	29
An explanation of the treatment	2	6
Do you know why you were asked to sign the informed consent?		
To protect your rights as a patient	0	0
Because it is a new treatment and can have unwanted effects	8	23
To free the Institution and the doctors of any responsibility in case something bad happens	10	29
To have access to free treatment	16	46
Did you read the document?		
Yes	20	57
No	15	43
If not explain why?		
I don't know how to read	11	31
The doctor's explanation was enough	9	26
If yes		
Once	8	23
More than once	7	20
At home	7	20
At the hospital	8	23
By myself	2	6
With my family	11	31
After reading the documented, your understanding about the study improved?		
Yes	9	26
No	6	17
Do you have any comments about the document?		
The document is too long	23	66
The document is difficult to understand	17	49
I could not understand it	17	49
I got bored and did not read it completely	10	29
The doctor explained it to me and i did not read it	11	31
It is a waste of time	12	34
If i don't get into the "protocol, i won't receive any treatment	16	46
I did not understood many of the words	17	49
Why did you decide to participate?		
To help others	0	0
Because the treatment will help me	30	86
To get better	30	86
To increase the knowledge about my disease	0	0
Because I don't have money to have any other treatment	21	60
Because my Doctor asked me and he knows what is good for me	25	71
Because someone in my family told me	5	14
Because the treatment was free	21	60
I don't know	3	8

A selection of the relevant questions and answers given by the patients are shown (the complete questionnaires are shown in the appendix). The patients and a family member were provided with the questionnaire. It was answered in a quiet room; although study personnel were not present in the room at the time, staff members were available outside to answer questions. When required, the questionnaire was read to the illiterate patients involved in the research study; otherwise, the patient's family member read out the questions and checked off the selected answer.

Some patients, provided answers not present in the questionnaire.

The document was read by only 57% of the patients, though the main reason for not reading the consent was illiteracy (31%). Three patients (8%) did not read the consent form because they considered that "the explanation their physicians provided was sufficient for them to decide to sign the document". Most of the patients read the document only once, outside of their doctor's office, at the hospital, and decided to participate in the study in less than an hour (data not shown, this question was asked directly to the patients). Thirty three patients decided to participate in the study with the help of someone from their family and only two patients read and signed the document on their own. Only eight of the patients who read the document believed that their understanding of the treatment proposal improved after reading the document (Table 2).

Each and every one of the patients who read the document thought that it was lengthy. Many patients considered that it was difficult to understand and that the document had many words of which they "didn't know the meaning". Almost half of the patients believed that signing the document was mandatory before they could receive treatment (as a "requirement", not as a right to know). Many thought that their doctor's explanation was easier to understand than the informed consent document. The reasons that were cited as the most important for enrolling in trials included: improving their health, having access to an otherwise inaccessible treatment, and because their doctor thought it was the best option for the patient (Table 2).

### Doctors

#### Participation and knowledge about the IC

Participation of physicians in the survey was limited, only twenty (out of 50 doctors involved in clinical research trials at the institution) answered the questionnaire. All participating doctors knew that their patients had to be informed about the treatment. All of them answered that the IC was a requirement of the hospital, the clinical research organisation, and/or the sponsors' international authorities and/or an ethical request for research. The requirement of the informed consent for conducting research in humans was acknowledged by all doctors; however, most of them were not sure if and where this requisite present in the current Mexican Health Law.

None of the doctors had ever participated in an informed consent design and/or suggested any changes to the sponsors, and all of them acknowledge the Declaration of Helsinki as the document that addresses ethical issues for research involving humans. (Table 3)

**Table 3:** Doctors' Responses

Questions	No	%
What is the purpose of the informed consent?		
To inform the patient about the treatment	20	100
A requirement of the hospital authorities	20	100
A requirement of the Mexican health authorities	0	0
A requirement of the international sponsors	20	100
A requirement of the Clinical Research Organizations (CRO)	20	100
Have you been involved in designing the informed consent?		
Yes	0	0
No	20	100
Are you familiar with the International regulations applied for developing the informed consent procedure?		
Yes	0	0
No	20	100
Is the IC a requirement present in the: Mexican LGS; Mexican Regulation for Research or is it an international request?)*		
Is present in the Mexican LGS	20	100
Is present in the Mexican Regulation of Research	0	0
It is an international requirement	20	100
It is present in all of them	0	0
Which of the following statements best describes purpose of the informed consent?		
A document required for biomedical research	15	75
Guidelines to prepare the informed consent	13	65
A document that will "protect" the physician/researcher in case of legal procedure	15	75
A document to emphasize the "shared" responsibility between doctors and patients	17	85
Not sure	5	25
Do you know what makes a patient vulnerable?		
Being a prisoner	20	100
Being poor	0	0
Being sick	0	0
Not having medical care	20	100
Do you think that the patients admitted to the hospital are vulnerable?		
Yes	3	15
No	17	85
Which of the following conditions may influence the informed consent procedure?		
Being the treating physician	0	0
If the patient is vulnerable	0	0
Conflict of interest of the physician	20	100
When conducting a clinical trial, are you involved in the patients' treatment before the trial?		
Never	0	0
Sometimes	1	5
Always	19	95
Did you receive financial compensation from the sponsor company (additional salary, trip, congresses)?		
Yes	17	85
No	3	15
Do you think there is a conflict of interest because of this?		
Yes	0	0
No	20	100
Do you think the patient understands the wording of the informed consent?		
Yes	7	35
No	13	65

The questionnaires were sent out by e-mail or were delivered in a sealed envelope. The answers were submitted anonymously. Three weeks after sending the questionnaire and if a response had not arrived; the physicians were contacted to see if they had sent their responses. Although 70 questionnaires were sent out, only 20 physicians responded.

* The Mexican Regulation for Research is the document that describes the IC document and tried to document. The purpose of this question was to learn about the doctors knowledge about the Mexican regulations, and if they had ever read the regulation.

To the specific question about "vulnerable population" the concept was only recognised for children, pregnant women, prisoners, and those patients living in areas with access to medical care. Economic educational characteristics, medical condition, situation and desperation for treatment may offer any hope were not acknowledge as features of a vulnerable population. Only 15% of the doctors thought that the population the hospital was vulnerable.

### Conflict of interest

Although the physicians conducting the clinical trials were also the treating doctors, they were unaware of possible conflicts of interest and did not consider that it interfered with the consent procedure (Table 3).

All the clinical researchers received gifts from the sponsors (lunches, trips to congresses, etc.), and in most cases (85%), financial compensation. These circumstances were not regarded as to influence their ethical behaviour, or to represent a conflict of interest. Finally, most of the doctors believed that the patient did not fully understand the informed consent document (Table 3).

Although questions were not made about moral principles and ethical behaviour, all of the responding doctors thought that the survey put in doubt their integrity as physicians (these arguments were made verbally, no written complaint was made).

### Consent forms

None of the ten consent forms reviewed had a uniform format; however, all had at the beginning of the document the name of the study, sponsors' name and address, along with the principal investigator's name. Most of the international clinical trials at the institution were made for marketing purposes, 10% Phase I, 30% Phase II, and 60% Phase III/IV (data not shown).

Although the studies did not have a uniform description, all the consent forms fulfilled the basic elements of the informed consents, as required by international ethical guidelines. The consent forms were 14 pages long on average (9–27 pages), and originally written in English (Table 4). Although the overall "technical" accuracy of the translation was reasonable, the use of scientific words and/or abbreviations of compounds were the rule; often the drugs to be studied were mentioned by their brand name. The presence of medical terms, language not used by non-medical individuals and words unfamiliar to the population that attended the institution were frequently used in the informed consent (Table 4). For a native Spanish speaking person, literal translation of the document was evident and the meaning of some phrases/paragraphs was difficult to understand. The average readability score of the ten documents was at an 8TH Grade level (4th-12th Grade) (Table 4).

**Table 4:** Informed Consent Evaluation

Item	Average Score (range)
Number of Consent Forms	10
International Requirement for IC	All 10/10
Number of pages	14.7 9–27
Original language of the CF	E
Accuracy of the translation*	8 6–9
Use of medical or scientific terms**	4.7 2–7
Language unfamiliar to non-medical individuals**	5 2–5
Language unfamiliar to average individual found at the institution**	5.9
Readability Score^&^	8 4–12

Qualitative evaluation of the Consent forms.

The Consent Forms were individually read and evaluated by a single rater and there is no inter-rater reliability data.

; a numerical value was assigned to some qualitative values using visual scales.

* 1 = poor accuracy ... 10 = good accuracy;

** 1 = few terms or words ... 10 = many terms or words.

Results represent average values and range, when applicable.

^&^Average value: Non-uniform wording was found; Consent Forms had wording with different readability scores.

The procedures to be applied during the trial were carefully described in the consent forms. Often, forms to request biological samples (tissue, blood, serum) and authorisations for every thing were included in the same document. In at least two studies, enrolment was conditioned to the collection of biological samples; one of them described a DNA analysis and the fact that the results would not be available to the patients (data not shown). Comprehension of the consequences of genetic material requests was not evaluated in this study.

Two sample Consent Forms were chosen to be reviewed by the ten individuals chosen to assess their comprehensibility. According to one of these individuals (the resident), the consent form was clear. The rest of the participants thought that the documents were lengthy, boring, and too complex to be understood. Most of the individuals needed to read the forms at least twice; however, when asked specific questions about the study, only four participants were able to describe correctly the purpose of the study (Table 5).

**Table 5:** Qualitative Assessment of Comprehensibility

Questions	Answers
Did you understand the purpose of the letter?	All The answers were yes
What was the study about?	6/10 were unable to describe the purpose of the study
Are there any words that you were unable to understand?	7/10 answered yes
Do you think that the document was clear?	Only the medical resident said yes
Were there any medical terms in the document?	10/10 said yes
How many times did you read the document	2/10 read it twice
What do you think about the length of the document	9/10 considered the document lengthy

The comprehensibility of selected consent form was qualitatively scrutinized with the help of ten individuals who had previously agreed to participate in the survey. Two nurses (N); two secretaries(S); three ambulance drivers (D); two social workers (SW); and one medical resident(R). Selected consent forms were given to read to these individuals after the sponsor's name and study drug(s) were blanked. Afterwards, the short questionnaire was handed out, and they were asked to answer all but the second question. Although, all answered that they understood the purpose of the study, when asked what it was about, only four answered correctly.

## Discussion

Public hospitals represent the principal setting for internationally sponsored research in Mexico; these institutions provide health care to a sector of the population that has neither access to social security or private medical attention. This work exemplifies the characteristics of both, patients and physicians in this scenery; while doctors are usually highly professionals (data not shown); most patients have the lowest income in the country. Furthermore, many of the patients included in this work were living in poverty and had a low literacy level (only twenty percent of the population had high school education or beyond).

In addition, these patients had been receiving treatment for some time; many of them had advanced disease (data not shown). Therefore, as is often the case in the setting of cancer trials for patients with advanced, eventually life ending disease; the chance of meaningful objective therapeutic benefit traditionally has been described as being quite low [41]. Consequently, even in developed countries there is a consensus that these patients may be considered vulnerable populations, and efforts to protect them from any time of exploitation should be mandatory [42,43].

It is particularly worrisome the complex setting in which enrolment of the patients into a research trial takes place; researchers are also the treating physicians, and often they have been the patients' doctor for a long period of time. In this situation, an invitation to a research trial may be interpreted by many patients to be an endorsement by their physicians [44].

The ethical consequences of the dual figure – Physician/researcher – that prevail in the clinical research field have been extensively addressed. The physician's obligation to treat patients in a way that will be most beneficial may put into question their research motives [46-50]. Whether or not the researcher should be regarded as a doctor, and whether or not the same obligations should apply to both doctors and researchers is a matter of debate. While some authors argue that they must share the same obligations, there are those who believe that clinical research and therapeutic medical practice are sufficiently distinct activities to require different ethical rules and principles [51,52].

An important aspect that should be addressed, and a reason of concern from a bioethical perspective worldwide, is clearly reviewed in this work: the presence of financial ties between pharmaceutical companies and Mexican oncologists. Supplemental income provided to researchers by pharmaceutical companies is common, and gifts as part of the relation with the industry are the rule. Although the situation clearly represents a conflict of interest, none of the physicians questioned recognized that this might jeopardize a patient's well being in favor of possible research success [32].

Also, economic influence of international pharmaceutical companies over physicians needs to be acknowledged. It is clear that doctors do not believe that gifts, salaries or trips jeopardize the ethics of their practice; however, making it a common practice may eventually be questionable [32].

The IC procedure is heralded as the principal mechanism to ensure that research takes place according to ethical principles. However, IC is a complex and somewhat idealised process [53]. In order that a valid informed consent is given, U.S. federal regulations specify eight pieces of information that must be disclosed to research participants, ethically valid informed consent demands more than just disclosure [54]. Research participants should also understand the essential disclosed information; moreover, according with O'Neill, consent is a positional attitude, a ritual that cannot be given by vulnerable populations [18,55].

The requirement to have an independent decision-making, during the IC, is unreal in the clinical setting. Many patients have diminished independence, particularly at times when they are most in need of medical care; therefore, it is important to open the bioethical view to the social and medical aspects that are an indivisible part of the sick [11-17]. In addition; anxiety, stress and the complex information is provided to participants in oncology clinical trials, requires higher levels of understanding, and often, patients' do not acknowledge the treatments offered are experimental [26,27].

If independent decision-making is to be encouraged, informed consents should be adapted according to the targeted population [45]. It is clear from the results presented in this paper that, in few patients, the information given in these consent forms allows them to give an independent and informed decision. The need to consider social and cultural background, when encouraging 'independence' and an equitable doctor-patient relationship, is of major importance; in some populations, like the one presented in this work, these actions could be perceived by the patients as abandonment, as Hedegecoe has argued [46].

The present work shows that neither the wording nor the lengths of current IC documents are useful in ensuring a comprehension of the nature of the research or to help the patients make an informed and individual decision [58]. In a recent proposal prepared by the American Society of Clinical Oncology, the creation of a 'central organisation' to oversee the clinical trials and consent forms has been suggested [59]. If this proposal is accepted, clinical researchers from different countries should be aware that the nature of the informed consent cannot be described in abstract.

The practice of IC has been driven by two different agendas: a legal one and a moral one [53]. In our experience, sponsoring companies approach informed-consent documents as compulsory legal regulatory requirements. We believe that clinical researchers and the Ethics' Committees should play an active role suggesting the required changes to the ICF and/or to the way the patients are *consented*; to guarantee that IC fulfills not only the legal-regulatory requirements, but that it is adjusted to the requirements of the targeted population [54,55].

Several authors have addressed different approaches to improve the patients' comprehension of experimental treatments; the time with the potential participant seems to be the best way to improve the understanding of the patients [55,60-62]. It is also important to acknowledge that these patients should be considered a vulnerable population and proper actions should be implemented to protect them, without depriving them from the benefits of experimental treatments. In the setting of developing countries, important considerations should be made regarding the informed consent. Even if the procedure follows the established guidelines of 'Good Clinical Practice' it may not accomplish its purpose when a vulnerable non-autonomous population is being consented.

We are aware of the study limitations; these findings may not be representative of all the settings within the country, in some clinical settings the populations have higher educational levels; however, most clinical trials are conducted in public hospitals where the average population may have similar characteristics to those described in this work.

### Recommendations

The need to redress the balance of the informed consent, so that the ethical process is simple, concentrates on the rights of individuals and is institutionally mandatory [55]. The use of short video capsules about patients' rights could be arranged in different waiting areas of the institutions; language should be respectful and simple according to the educational level of the average population. The exposure to these materials could make it simpler for them to recognise when they are being invited to be part of a research trial, sometimes long before other types of treatments have failed.

In addition, researchers should be aware that the informed consent is a procedure needed to establish a respectful and ethical relation between doctors and patients.

## Conclusion

There is no simple answer to the inevitable question of 'how valid is the informed consent for these patients?' But it is clear that some adjustments should be implemented, accordingly with the characteristics' described in this population.

## Competing interests

The author(s) declare that they have no competing interests.

## Authors' contributions

Dr. Emma Verástegui, designed, conducted and wrote the manuscript. Part of the work was presented by Dr. Verástegui during her MSc. at the London School of Economics and Political Sciences.

## Pre-publication history

The pre-publication history for this paper can be accessed here:



## Supplementary Material

Additional file 1 The documents presented here were originally written in Spanish; therefore, were not the actual survey documents. To improve readability in English, minor editorial changes were made. The participants read and answered the questionnaire in Spanish. Invitation letter. The data provided represents the translated invitation letter provided to the patients. Click here for file

Additional file 2 The documents presented here were originally written in Spanish; therefore, were not the actual survey documents. To improve readability in English, minor editorial changes were made. The participants read and answered the questionnaire in Spanish. Patient's questionnaire. The data provided represent the translated questionnaires given to the patients. Answers given are also included, and the percentages for each response. Click here for file

Additional file 3 The documents presented here were originally written in Spanish; therefore, were not the actual survey documents. To improve readability in English, minor editorial changes were made. The participants read and answered the questionnaire in Spanish. Doctors' questionnaire. The data provided represent the translated questionnaires given to the doctors. Answers given are also included, and the percentages for each response. Click here for file

Additional file 4 The documents presented here were originally written in Spanish; therefore, were not the actual survey documents. To improve readability in English, minor editorial changes were made. The participants read and answered the questionnaire in Spanish. (part A) Analysis of the consent forms. Individual evaluation of each individual consent forms. (part B) Comprehensibility. Comprehensibility of the consent forms. The qualitative analysis was done with the help of ten different individuals. Click here for file
