# Supplementary material for: Consenting of the vulnerable: the informed consent procedure in advanced cancer patients in Mexico
Source: BMC Med Ethics. 2006 Dec 13;7:13. doi: 10.1186/1472-6939-7-13 (PMC1764745; doi:10.1186/1472-6939-7-13)
Supplement: Additional file 1 — The documents presented here were originally written in Spanish; therefore, were not the actual survey documents. To improve readability in English, minor editorial changes were made. The participants read and answered the questionnaire in Spanish. Invitation letter. The data provided represents the translated invitation letter provided to the patients. [file 1472-6939-7-13-S1.doc]

Additional file 1

Instituto Nacional de Cancerologia

Informed consent survey: invitation letter[[1]](#footnote-2)

Dear sir/madam:

We would like to invite you to participate in a survey that we are conducting.

The survey is designed for those patients who have been invited to participate in a research trial (a “protocol”). We would like to learn about the processes of this “invitation”, who invited you? How were you invited? What type of material was given to you and why did you accept?

The information gathered with the survey will allow us to find out if there is something during the process that requires adjustment and needs to be suggested to the authorities. Also, if there is need for improvement in any of the steps.

If you accept to answer the questionnaire, please reply truthfully. The survey will not require you to provide your name. If you are not able to read or write, please let us know so we can help you.

The results of the survey may be published, but your name will never appear; your participation is voluntary and, if you don’t want to answer the questions, there won’t be any change in your medical attention.

The survey was presented to and approved by the Hospital Ethics Committee and they decided that you don’t need to sign a consent document. However, you may want to think or talk with some one in your family before you decide to answer the questions.

The survey has ten questions, and you may choose more than one answer if needed or write-in any other answer. There are no right or wrong answers; we want to understand some of the reasons that made you enter the trial. Although we won’t be present when you answer the questions, if you need any assistance we will be in the next room.

Thank you for your participation

Sincerely yours

Dr. Emma Verastegui

56280400-ext 383

1. The content of the letter was explained to the patients as they were reading it. [↑](#footnote-ref-2)
