# Supplementary material for: Consenting of the vulnerable: the informed consent procedure in advanced cancer patients in Mexico
Source: BMC Med Ethics. 2006 Dec 13;7:13. doi: 10.1186/1472-6939-7-13 (PMC1764745; doi:10.1186/1472-6939-7-13)
Supplement: Additional file 2 — The documents presented here were originally written in Spanish; therefore, were not the actual survey documents. To improve readability in English, minor editorial changes were made. The participants read and answered the questionnaire in Spanish. Patient's questionnaire. The data provided represent the translated questionnaires given to the patients. Answers given are also included, and the percentages for each response. [file 1472-6939-7-13-S2.doc]

Additional file 2

Patients’ questionnaire

1. For how long have you been treated at this hospital?

| Less than one year | 6 | 17% |
| --- | --- | --- |
| 2-5 years | 21 | 60% |
| More that 5 years | 8 | 23% |

1. Do you know how to write and read?

| Yes | 24 | 69% | No | 11 | 31% |
| --- | --- | --- | --- | --- | --- |

For how long did you attend school?

| Never went to the school | 11 | 31% |
| --- | --- | --- |
| I did not finish grade school | 10 | 29% |
| I finished grade school | 7 | 20% |
| I did not finish high school | 4 | 11% |
| I finished high school | 3 | 9% |
| I went to College | 0 | 0 |
| I finished College | 0 | 0 |
| I have graduate school education | 0 | 0 |

1. Who invited you o participate in the clinical trial?

| My treating doctor | 30 | 86% |
| --- | --- | --- |
| A “new” doctor | 4 | 11% |
| A resident | 1 | 3% |
| A nurse | 0 | 0 |
| A social worker | 0 | 0 |
| Other | 0 | 0 |

1. Were you asked to read and sign an informed consent

| Yes | 35 | 100% | No | 0 | 0 |
| --- | --- | --- | --- | --- | --- |

5. Do you know what a consent form is?

| A document part of the protocol | 23 | 65% |
| --- | --- | --- |
| A document i have to sign before i can be admitted to the study | 10 | 29% |
| An explanation of the treatment | 2 | 6% |
| A document that informs me about a new way to treat my cancer and gives me the right to make a decision | 0 | 0 |

6. Do you know why you were asked to sign the informed consent?

| To protect your rights as a patient | 0 | 0 |
| --- | --- | --- |
| To be sure that you know that it is an experimental treatment | 5 | 14% |
| Because it is a new treatment and can have unwanted effects | 8 | 23% |
| Ii is a requirement for the study | 3 | 9% |
| To share the responsibilities for the treatment (the Institute and the patients) | 4 | 11% |
| To free the Institution and the doctors of any responsibility in case something bad happens. | 10 | 29% |
| To get free treatment | 16 | 46% |

7. Did you read the document?

| Yes | 20 | 57% | No | 15 | 43% |
| --- | --- | --- | --- | --- | --- |

If not, explain why?

| I don’t know how. to read | 11 | 31% |
| --- | --- | --- |
| My doctor explained it and that was enough | 3 | 8% |

If not (and you don’t know to read)

| How did you Sign?  I used my fingerprint  I know how to put my name | 1  10 | 2  29 |
| --- | --- | --- |
| Did someone form you family read the document for you?  Yes  No | 8  3 | 23  8 |

If yes

| Once | 11 | 31% |
| --- | --- | --- |
| More than once | 9 | 26% |
| At home | 9 | 26% |
| At the hospital | 11 | 31% |
| By yourself | 2 | 6% |
| With someone else from my family | 16 | 46% |
| With the doctor | 0 | 0 |
| With someone else from the hospital | 2 | 6% |

8. After reading the document, did your understanding about the study improve?

| Yes | 8 | 23% | No | 12 | 34% |
| --- | --- | --- | --- | --- | --- |

9. Do you have any comments about the document?

| The document is too long | 20 | 57% |
| --- | --- | --- |
| The document is difficult to understand | 17 | 49% |
| I could not understand it | 17 | 49% |
| I got bored and did not read it completely | 10 | 29% |
| The doctor explained it to me and i did not read it | 11 | 31% |
| It is a waste of time | 12 | 34% |
| If I don’t get into the “protocol, I won’t receive any treatment | 16 | 46% |
| I did not understood many words | 18 | 51% |

10. Why did you decided to participate?

| To help others | 0 | 0 |
| --- | --- | --- |
| Because the treatment will help me | 30 | 86% |
| To get better | 30 | 86% |
| To increase the knowledge about my disease | 0 | 0 |
| Because I don’t have money to have any other treatment | 21 | 60% |
| Because my Doctor asked me and he knows what is good for me | 25 | 71% |
| Because someone in my family told me | 5 | 14% |
| Because the treatment was free | 21 | 60% |
| I don’t know | 3 | 8% |

The information present in the results section, represent a summary of these results. To make the results easier to be read some changes were made. Some times the patients provided additional information to the question asked, a summary of it is provided in the results section.
