# Supplementary material for: Consenting of the vulnerable: the informed consent procedure in advanced cancer patients in Mexico
Source: BMC Med Ethics. 2006 Dec 13;7:13. doi: 10.1186/1472-6939-7-13 (PMC1764745; doi:10.1186/1472-6939-7-13)
Supplement: Additional file 3 — The documents presented here were originally written in Spanish; therefore, were not the actual survey documents. To improve readability in English, minor editorial changes were made. The participants read and answered the questionnaire in Spanish. Doctors' questionnaire. The data provided represent the translated questionnaires given to the doctors. Answers given are also included, and the percentages for each response. [file 1472-6939-7-13-S3.doc]

Additional file 3

Doctors’ questionnaire

1. What is the purpose of the informed consent?

| To inform the patient about the treatment | 20 | 100% |
| --- | --- | --- |
| A requirement of the sponsors’ country health authorities | 20 | 100% |
| A requirement of the hospital authorities | 20 | 100% |
| A requirement of the Mexican health authorities | 0 | 0 |
| A requirement of the international sponsors | 20 | 100% |
| A requirement of the Clinical Research Organisations (CRO) | 20 | 100% |

**2. Have you been involved in the informed consent design?**

| Yes | 0 | 0 | No | 20 | 100% |
| --- | --- | --- | --- | --- | --- |

3. Are you familiar with the International regulations applied for the informed consent procedure?

| Yes | 20 | 100% | No | 0 | 0 |
| --- | --- | --- | --- | --- | --- |

4. Can you mention any international document that addresses ethical issues for biomedical research involving humans?

| Helsinki Declaration | 20 | 100% |
| --- | --- | --- |

5. Are you familiar with Mexican General Law of Health[[1]](#footnote-2)?

| Yes | 20 | 100% | No | 0 | 0 |
| --- | --- | --- | --- | --- | --- |

6. Is the Written Informed Consent a Requirement for research involving humans in the Mexican Law?

| Yes | 15 | 75% | No | 5 | 25% |
| --- | --- | --- | --- | --- | --- |

Have you read the local (Mexican) guidelines about the informed consent?

| Yes | 15 | 75% | No | 5 | 25% |
| --- | --- | --- | --- | --- | --- |

What of the following statements best describes the contents of the Mexican General Law of Health regarding the informed consent?

| A document required for biomedical research | 15 | 75% |
| --- | --- | --- |
| Guidelines for preparation of the informed consent | 13 | 65% |
| A document that will "protect" the physician/researcher in case of legal procedures | 15 | 75% |
| A document to emphasize the "shared" responsibility between doctors and patients | 17 | 85% |
| Not sure | 5 | 25% |

Are you familiar with any other Mexican Document that talks about the Informed consent?

| Yes | 0 | 0 | No | 20 | 100% |
| --- | --- | --- | --- | --- | --- |

**7. Do you know what makes a patient being vulnerable?**

| Being a prisoner | 20 | 100% |
| --- | --- | --- |
| Being poor | 0 | 0 |
| Being sick | 0 | 0 |
| Not having access to medical care | 20 | 100% |

Do you think that the patients admitted to the hospital are vulnerable?

| Yes | 3 | 15% | No | 17 | 85% |
| --- | --- | --- | --- | --- | --- |

8. Which of the following conditions may influence the informed consent procedure?

| Being the treating physician | 0 | 0 |
| --- | --- | --- |
| If the patient is vulnerable | 0 | 0 |
| Conflict of interest of the physician | 20 | 100% |

When conducting a clinical trial, are you involved in the patients’ treatment before the trial?

| Never | 0 | 0 |
| --- | --- | --- |
| Some times | 1 | 5% |
| Always | 19 | 95% |

Is there any condition for you as a doctor to request the informed consent?

| To work in the Institution | 20 | 100% |
| --- | --- | --- |
| To have “good” ethical principles | 20 | 100% |
| To Follow the Good Clinical Practices^24^ | 20 | 100% |
| To be free of any conflict of interests | 20 | 100% |

9. Do you get any compensation from the sponsor company?

| Yes | 17 | 85% | No | 3 | 15% |
| --- | --- | --- | --- | --- | --- |

If yes, please describe the type of compensation

| Additional income | 17 | 85% |
| --- | --- | --- |
| invitation to dinner/lunch | 17 | 85% |
| invitation to medical conferences | 20 | 100% |
| invitation to the American Society of Clinical Oncology Meeting | 20 | 100% |

Do you think you have a conflict of interest because of this?

| Yes | 0 | 0 | No | 20 | 100% |
| --- | --- | --- | --- | --- | --- |

1. **Do you think the patient understands the wording of the informed consent?**

| Yes | 7 | 35% | No | 13 | 65% |
| --- | --- | --- | --- | --- | --- |

The information present in the results section, represent a summary of these results. To make the results easier to be read some changes were made. Often the doctors provide additional information to the question asked, a summary is provided in the results section.

1. Mexican Health Legislation [↑](#footnote-ref-2)
