# Supplementary material for: Consenting of the vulnerable: the informed consent procedure in advanced cancer patients in Mexico
Source: BMC Med Ethics. 2006 Dec 13;7:13. doi: 10.1186/1472-6939-7-13 (PMC1764745; doi:10.1186/1472-6939-7-13)
Supplement: Additional file 4 — The documents presented here were originally written in Spanish; therefore, were not the actual survey documents. To improve readability in English, minor editorial changes were made. The participants read and answered the questionnaire in Spanish. (part A) Analysis of the consent forms. Individual evaluation of each individual consent forms. (part B) Comprehensibility. Comprehensibility of the consent forms. The qualitative analysis was done with the help of ten different individuals. [file 1472-6939-7-13-S4.doc]

Additional file 4 (part A)

Analysis of the consent forms

|  | 001 | 002 | 003 | 004 | 005 | 006 | 007 | 008 | 009 | 010 | Average |
| --- | --- | --- | --- | --- | --- | --- | --- | --- | --- | --- | --- |
| Basic elements of informed consent^34^ |  |  |  |  |  |  |  |  |  |  |  |
| Number of pages | 15 | 27 | 15 | 10 | 17 | 9 | 13 | 20 | 11 | 10 | 14.7pgs |
| Original language of the CF | E | E | E | E | E | E | E | E | E | E |  |
| Accuracy of the translation* | 8 | 8 | 9 | 8 | 9 | 7 | 6 | 9 | 9 | 7 | 8 |
| Use of medical or scientific terms** | 3 | 7 | 5 | 5 | 4 | 2 | 6 | 5 | 5 | 3 | 4.7 |
| Language not used by non-medical individuals** | 5 | 6 | 6 | 6 | 6 | 2 | 5 | 6 | 6 | 2 | 5 |
| Language not used by average individual attending the institution** | 6 | 6 | 6 | 6 | 6 | 6 | 5 | 6 | 6 | 6 | 5.9 |
| Readability score | 8^th^  ^Grade^ | 12^th^  ^Grade^ | 12^th^  ^Grade^ | 8^th^  ^Grade^ | 8^th^  ^Grade^ | 4^th^  ^Grade^ | 8^th^  ^Grade^ | 8^th^  ^Grade^ | 8^th^  ^Grade^ | 6^th^  ^Grade^ | 8^th^  ^Grade^ |

Analogue visual scales used to give a numerical value to the answers*

| POOR  1 | 2 | 3 | 4 | 5 | 6 | 7 | 8 | 9 | GOOD  10 |
| --- | --- | --- | --- | --- | --- | --- | --- | --- | --- |

**

| FEW  1 | 2 | 3 | 4 | 5 | 6 | 7 | 8 | 9 | MANY  10 |
| --- | --- | --- | --- | --- | --- | --- | --- | --- | --- |

Qualitative evaluation of the Consent forms.

The Consent Forms were individually read and evaluated by a single rater and there is no inter-rater reliability data.

A numerical value was assigned to some qualitative values using visual scales.

* 1 = poor accuracy … 10 = good accuracy;

** 1 = few terms or words … 10 = many terms or words.

Results represent average values and range, when applicable.

^&^ Average value: Non-uniform wording was found; Consent Forms had wording with different readability scores.

Additional file 4 (part B)

Comprehensibility

|  | D | S | N | SW | D | N | SW | D | S | R |
| --- | --- | --- | --- | --- | --- | --- | --- | --- | --- | --- |
| Did you understand the purpose of the letter? | Y | Y | Y | Y | Y | Y | Y | Y | Y | Y |
| What was the study about? | ? | ? | OK | OK | ? | OK | ? | ? | ? | OK |
| Are there any words that you were not able to understand? | Y | Y | N | N | Y | Y | Y | Y | Y | N |
| Do you think that the document was clear? | N | N | N | N | N | N | N | N | N | Y |
| Were there any medical terms in the document? | Y | Y | Y | Y | Y | Y | Y | Y | Y | Y |
| How many times did you read the document? | 2 | 2 | 2 | 2 | 2 | 1 | 2 | 2 | 2 | 1 |
| What do you think about the length of the document  Too short =Sh  Lengthy =L | L | L | L | L | L | L | L | L | L | OK |

The comprehensibility of the consent form was qualitatively scrutinized with the help of ten individuals: two nurses (N); two secretaries(S); three ambulance drivers (D); two social workers (SW); and one medical resident(R). Selected consent forms were given to read to these individuals after the sponsor’s name and study drug(s) were blanked. A standard set of questions were asked after reading the chosen consents.
